# Efficacy of two distinct ethanol-based hand rubs for surgical hand disinfection – a controlled trial according to prEN 12791

**DOI:** 10.1186/1471-2334-5-17

**Published:** 2005-03-22

**Authors:** Günter Kampf, Christiane Ostermeyer

**Affiliations:** 1 Bode Chemie GmbH & Co., Scientific Affairs, Melanchthonstr. 27, 22525 Hamburg, Germany; 2 Institut für Hygiene und Umweltmedizin, Ernst-Moritz-Arndt Universität Greifswald, Walther-Rathenau-Str. 49a, 17489 Greifswald, Germany; 3 Bode Chemie GmbH & Co., Microbiology, Melanchthonstr. 27, 22525 Hamburg, Germany

## Abstract

**Background:**

Aim of the study was to determine the efficacy of two distinct ethanol-based hand rubs for surgical hand disinfection in a controlled cross-over trial according to prEN 12791.

**Methods:**

20 subjects were included. Hands were washed for 1 min with soap. The bacterial prevalue was obtained by rubbing finger tips in TSB for 1 min. Then, each subject treated the hands with the reference procedure (n-propanol, 60% v/v) or the product (Sterillium^®^ Rub, based on 80% ethanol; Avagard, based on 61% ethanol and 1% chlorhexidine gluconate) which were all applied in 3 to 4 portions each of 3 ml for a total of 3 min. Bacterial postvalues (immediate effect) were taken from one hand, the other hand was gloved for 3 h. After gloves were taken off the second postvalue was taken for the assessment of a sustained effect.

**Results:**

Bacterial pre-values were between 4.38 ± 0.66 and 4.46 ± 0.71. Sterillium^® ^Rub achieved the required immediate (mean log_10_-reduction of 2.59 ± 1.19) and sustained effect (1.73 ± 1.08) compared with the reference treatment (immediate effect: 2.58 ± 1.16; sustained effect: 1.67 ± 0.96). Avagard, however, did not achieve the required immediate (1.82 ± 1.40) and sustained effect (1.41 ± 1.08) in comparison to the reference disinfection (immediate effect: 2.98 ± 0.90; sustained effect: 2.56 ± 1.17; p < 0.01; Wilcoxon test).

**Conclusion:**

Based on our data, Sterillium^® ^Rub can be regarded to be effective for surgical hand disinfection, but Avagard can not. The addition of 1% chlorhexidine gluconate to 61% ethanol (w/w) did not outweigh an ethanol concentration of 80% (w/w).

## Background

The new CDC guideline on hand hygiene has indicated that the efficacy of alcohols is superior to many other active agents such as chlorhexidine gluconate or povidone iodine, also on the resident hand flora [1]. Alcohol-based hand rubs are commonly used for surgical hand disinfection in Europe [2]. Their *in vivo *efficacy is usually tested according to prEN 12791 under practical conditions against a reference treatment [3]. This means that a product shall not be significantly less effective compared to a reference alcohol after 0 and 3 h (gloved hand). This test method is well suitable to discriminate the efficacy of various types of preparations based on different active agents [4]. To our knowledge, the efficacy of preparations for surgical hand disinfection based on different concentrations of ethanol has never been compared according to prEN 12791. The aim of this study was to evaluate the efficacy of two ethanol-based hand rubs for surgical hand disinfection, Sterillium Rub (80% ethanol, w/w) and Avagard (61% ethanol w/w, and 1% chlorhexidine gluconate) according to prEN 12791.

## Methods

Twenty subjects were included for each of two experiments. Hands were pre-washed with soap for 1 min. The bacterial prevalue was obtained by rubbing finger tips in tryptic soy broth (TSB) for 1 min. Afterwards, each subject treated the hands with the reference alcohol (n-propanol, 60% v/v) or the product. For the reference disinfection, n-propanol was applied in 3 to 4 portions each of 3 ml in order to keep the skin moist for a total of 3 min. Sterillium Rub™ based on 80% w/w ethanol and Avagard based on 61% w/w ethanol and 1% chlorhexidine gluconate were also applied in 3 to 4 portions in order to keep the skin moist for a total of 3 min. Bacterial postvalues (immediate effect) were taken from one hand by rubbing finger tips in TSB containing neutralizers (3% Tween 80, 3% lecithin, 0.1% histidine, and 0.1% cysteine) for 1 min, the other hand was gloved for 3 h. After gloves were taken off the second postvalue was taken by rubbing finger tips in TSB for 1 min for the assessment of a sustained effect. The bacterial concentration in the sampling fluid was determined by serial dilution and surface culture. The differences between the log_10 _pre- and postvalues were calculated individually for each subject [5]. Means of these differences were analyzed with the Wilcoxon matched-pairs signed-ranks test [6].

## Results

Sterillium Rub™ was found to be equally effective as the reference alcohol both in the immediate effect and after 3 h. The difference of the mean bacterial reduction at 0 h and 3 h between the reference treatment and Sterillium Rub™ was not significant (p > 0.1; Wilcoxon matched-pairs rank test; Table 1).

**Table 1:** Mean log_10_-reduction (RF) ± s.d. of Sterillium Rub™ (3 min) and Avagard in comparison to the reference alcohol (60% v/v n-propanol; 3 min) for surgical hand disinfection according to prEN 12791.

Product	Pre-value	0 h		3 h	
		mean RF	p-value	mean RF	p-value
Sterillium Rub	4.39 ± 0.83	2.59 ± 1.19	> 0.1	1.73 ± 1.08	> 0.1
Reference treatment	4.44 ± 0.90	2.58 ± 1.16		1.67 ± 0.96	

Avagard	4.46 ± 0.71	1.82 ± 1.40	0.009	1.41 ± 1.08	0.008
Reference treatment	4.38 ± 0.66	2.98 ± 0.90		2.56 ± 1.17	

Avagard was found to be less effective than the reference alcohol in both the immediate effect (0 h) and after 3 h. The difference of the mean bacterial reduction between the reference treatment and Avagard was significant at 0 h (p = 0.009) and 3 h (p = 0.008; Table 1).

## Discussion

Sterillium Rub™ was found to meet the requirements of prEN 12791 (version 1997) on the bactericidal efficacy for a surgical hand rub, but Avagard did not. The reason is probably a too low concentration of ethanol (61% w/w) in Avagard [7]. It has been shown earlier that ethanol at a concentration of 60% is far less effective against the resident hand flora than ethanol at 80% or more [8,9]. In addition, chlorhexidine gluconate at 1% in Avagard did not compensate for the low efficacy of 61% w/w ethanol. Even after 3 hours, there was no sustained effect under the gloved hand which raises doubts on the justification of this agent in the formulation.

This finding is in line with previously reported data. The efficacy of ethanol-based hand rubs on the resident hand flora varies considerably depending mainly on the concentration of the active agent. An immediate effect of 1.0 to 1.32 log_10_-reduction has been described with 70% w/w ethanol, a better effect of the resident skin flora can be found with ethanol at 85% w/w (mean reduction: 2.1 to 2.5 log_10_-steps) or 95% w/w (mean reduction: 2.1 log_10_-steps) [7]. The combination of 61% ethanol with 1% chlorhexidine gluconate has been described earlier to have superior bactericidal efficacy compared with an antimicrobial liquid soap based on 4% chlorhexidine, especially after 5 and 21 days use [10]. In another report the combination of 61% ethanol with 1% chlorhexidine was significantly more effective than 4% chlorhexidine soap on day 1 and 2 but not on day 5 [11] which is to some extent controversial to the data derived from the other study. In general, a better efficacy should be expected with an alcohol-based leave-on preparation containing chlorhexidine gluconate compared with a chlorhexidine-containing rinse-off preparation.

Alcohols without the addition of non-volatile agents such as quaternary ammonium compounds or chlorhexidine gluconate are regarded to have no sustained efficacy [1]. It is quite difficult to clearly define a sustained activity in surgical hand antisepsis. In the new CDC guideline, "persistent" activity is defined as the prolonged or extended antimicrobial activity that prevents or inhibits the proliferation or survival of microorganisms after application of the product [1]. But it remains unclear how such a persistent effect can be determined. According to the European norm prEN 12791 (version 1997) on products for surgical hand disinfection, a preparation has sustained efficacy if the mean RF is not significantly lower after 3 h, compared with the reference treatment. This reference treatment itself leads to a mean bacterial density on the hands which is usually significantly lower after 3 h compared to baseline [12] which can be described as a sustained efficacy. A persistent efficacy was defined as an efficacy after 3 hours which is significantly superior compared with the reference treatment regardless of the presence of a non-volatile active agents [5]. This definition was based on the knowledge that the reference alcohol does not contain any non-volatile active agent. A preparation with a non-volatile active agent such as chlorhexidine gluconate, however, may have an additional effect in comparison to the reference treatment. But following this definition, a preparation based on 85% ethanol (w/w) without any non-volatile active agent such as chlorhexidine gluconate has been described to have persistent activity [9]. In the new version of prEN 12791 (2003), the terminology has been changed. Now the term "sustained effect" describes the formerly "persistent effect" which may lead to some confusion. It would certainly be helpful to clearly define scientific terms and appropriate requirements in surgical hand disinfection which, ideally, are accepted worldwide [13].

The potential benefit of chlorhexidine gluconate is thought to be a prolonged effect. Repeated application is thought to increase the antimicrobial activity on the resident hand flora. If chlorhexidine gluconate is used in a "leave-on" preparation like Avagard it can be expected that the non-volatile active agent chlorhexidine gluconate remains on the skin and will continue to have antimicrobial activity. In our study we were not able to show that such an effect can be measured after a single application. In addition, permanent exposure to chlorhexidine salts has been shown to lead to adaptation or even resistance. Exposure of *Pseudomonas aeruginosa *to 5 mg/L chlorhexidine diacetate over a period of 12 days was able to increase the minimal inhibitory concentration (MIC) from ≤ 10 mg/L to 70 mg/L [14]. A similar observation was made after exposure of six strains of *Pseudomonas stutzeri *to gradually increasing concentrations of chlorhexidine diacetate which led to an increase of the MIC from 2.5 to 50 mg/L after 12 days [15]. Adaptation was also found with *Streptococcus sanguis *strains which were exposed to variable concentrations of chlorhexidine over a period of 10 weeks resulting in an increase of the MIC from 16 mg/L to up to 128 mg/L [16]. The resistance which has been developed on permanent exposure to chlorhexidine has been described to be stable and to include cross-resistance to other antiseptic agents (like triclosan or benzalkonium chloride) and antibiotics (like gentamicin, ampicillin, and erythromycin) [15]. Although this effect has to our knowledge not been reported with resident skin bacteria, it nevertheless underlines the potential of chlorhexidine gluconate once bacteria are permanently exposed to sub-lethal concentrations of the agent. If even antibiotic resistance can emerge by permanent exposure to chlorhexidine gluconate the potential benefit should be substantial to justify the addition of chlorhexidine gluconate in a surgical hand rub preparation from our point of view.

It is known that chlorhexidine salts are difficult to neutralize in experimental settings which may lead to false favorable results [17-20]. In one study, there was no effect at all against enterococci including VRE if neutralization of remaining chlorhexidine was ensured after the exposure time [21]. In the present study, neutralization of residual chlorhexidine was achieved after the exposure time which may be the explanation for the lacking effect after 3 hours.

In Europe, the efficacy of alcohol-based hand rubs for surgical hand disinfection is assessed using prEN 12791 [3]. The test principle is the cross-over evaluation with a reference alcohol (n-propanol 60%, v/v) which has been shown to have the best efficacy on the resident hand flora together with a "within-subject-comparison" of the bacterial reductions [8]. In addition, the test method has been described to yield reproducible results [22]. In the US, hand antiseptics are usually evaluated according to the test method published in the tentative final monograph for healthcare antiseptic products [23]. This test method is designed for rinse-off preparations. It does not include a reference treatment in the test on volunteers, but a preparation has to fulfill certain minimum requirements at various test days with higher requirements after 5 days [23]. This test philosophy is hard to understand and to justify since a patient who is treated on a Monday should have the same level of safety compared with the patient who is treated on a Friday. Inclusion of a reference treatment can be regarded to be superior since tests are done on the resident hand flora which may vary considerably in number and composition of bacterial species. The efficacy of a test preparation has to be equal to a suitable reference procedure at any time point providing the same level of safety for any patient. Only with inclusion of a reference treatment a true comparison of the efficacy between preparations can be achieved [4].

Alcohol-based hand rubs have been shown to have a better antimicrobial efficacy on both the transient and resident hand flora [2,7]. That is why is has been recommended in the new CDC guideline on hand hygiene that they may well be used for surgical hand disinfection [1] although it remains unclear if the use of preparations with a higher effect on the hand flora has an additional impact on the incidence of surgical site infections [24]. But another benefit has also been described: The use of a well formulated alcohol-based hand rub can improve the skin conditions of the surgeons resulting in significantly less skin dryness and significantly less skin irritation once they have changed from an antimicrobial soap to a well formulated alcohol-based hand rub [24]. Apart from the efficacy of a preparation, the dermal tolerance should also be considered [25].

## Conclusion

A high concentration of ethanol (80% w/w) was found to be effective on the resident hand flora after 0 and 3 hours, a lower concentration of ethanol (61% w/w), however, was not sufficiently effective if tested according to prEN 12791. The addition of 1% chlorhexidine gluconate to the 61% ethanol did not provide a substantial improvement of the bactericidal efficacy after 3 hours.

## Competing interests

Both authors are paid employees of Bode Chemie GmbH & Co., Hamburg, Germany.

## Authors' contributions

GK designed the study, analysed and interpreted the data. CO coordinated the study and acquired the data. Both authors drafted and revised the article.

## Pre-publication history

The pre-publication history for this paper can be accessed here:


